# Addition of quantitative imaging parameters to visual analysis improves the accuracy of PSMA PET/CT for the local staging of primary prostate cancer

**DOI:** 10.1007/s00259-025-07757-3

**Published:** 2026-02-13

**Authors:** Maarten L. Donswijk, Rosemarijn H. Ettema, Maurits Wondergem, Zing Cheung, Henk G. van der Poel, Daniela E. Oprea-Lager, André N. Vis

**Affiliations:** 1 Department of Nuclear Medicine, The Netherlands Cancer Institute - Antoni van Leeuwenhoek, Amsterdam, The Netherlands; 2 Department of Urology, Amsterdam University Medical Centres Amsterdam, Amsterdam, The Netherlands; 3 Prostate cancer network, Amsterdam, The Netherlands; 4 Department of Urology, St. Antonius Hospital, Nieuwegein, The Netherlands; 5 Department of Radiology and Nuclear Medicine, University Medical Centre Utrecht, Utrecht, The Netherlands; 6 Department of Nuclear Medicine, Noordwest Ziekenhuisgroep, Alkmaar, The Netherlands; 7 Department of Urology, The Netherlands Cancer Institute - Antoni van Leeuwenhoek, Amsterdam, The Netherlands; 8 Department of Medical Imaging, Radboud University Medical Center, Nijmegen, The Netherlands

**Keywords:** Prostate cancer, Positron emission tomography, Prostate-Specific membrane antigen, Extraprostatic extension, Quantitative parameters

## Abstract

**Purpose:**

Prostate-specific membrane antigen (PSMA) PET/CT is recognized as the most accurate imaging modality for staging of patients with intermediate and high-risk prostate cancer (PCa). PSMA PET/CT has also shown potential in the local (T) staging of primary PCa. The purpose of this study was to explore the value of quantitative PSMA PET/CT parameters in addition to the standard visual assessment for local T-stage classification in a large single-center retrospective cohort.

**Methods:**

Sequential intermediate- and high-risk primary PCa patients who underwent staging PSMA PET/CT prior to robot-assisted radical prostatectomy were included. Visual assessment of T-stage (miT-stage) was performed alongside quantitative analysis of PSMA PET/CT parameters, including SUVmax, SUVpeak, tumor volume (PSMA-vol), total lesion PSMA expression (PSMA-TL), and tumor longest capsule contact (LCC). The pathological tumor stage derived from radical prostatectomy specimens served as the reference standard. Univariable and multivariable logistic regression analyses were performed to develop clinical risk models for predicting pT3-stage disease.

**Results:**

A total of 223 evaluable patients with PSMA-positive primary PCa were included. Univariable analyses of individual imaging parameters yielded AUCs of 0.53–0.63 for pT3a, 0.64–0.74 for ≥ pT3b, and 0.59–0.69 for overall ≥ pT3-stage. In multivariable analyses, LCC was the sole independent predictor for pT3a stage; miT-stage, LCC, and PSMA-vol were independent predictors for ≥ pT3b-stage; and LCC together with PSMA-vol were independent predictors for overall ≥ pT3-stage. Clinical risk models incorporating these predictors achieved AUCs of 0.62 for pT3a, 0.79 for ≥ pT3b, and 0.70 for ≥ pT3-stage.

**Conclusion:**

Quantitative parameters derived from PSMA PET/CT scans provide additional diagnostic accuracy for detecting extraprostatic tumor extension, particularly for ≥ pT3b-stage disease, outperforming visual assessment (miT-stage) alone.

## Introduction

Prostate cancer (PCa) is the most prevalent malignancy among middle-aged and older men [1]. Accurate local staging (T-stage) at initial diagnosis is critical for guiding therapeutic strategies, including surgical planning—such as nerve-sparing approaches during radical prostatectomy—and for optimizing external beam radiotherapy. Curative treatment outcomes are more likely when the disease remains confined to the prostate gland (T-stage ≤ 2).

Prostate-specific membrane antigen (PSMA) positron emission tomography/computed tomography (PET/CT) is increasingly used for initial staging of patients with intermediate and high-risk primary PCa, as well as in those with recurrent disease after treatment with curative intent [2–4]. While PSMA-PET is an adequate modality for the staging of patients at risk for metastases, it may also be applied for the assessment of the local tumour extent of primary PCa.

In a previous large, multicentre study of patients with intermediate and high-risk PCa using both ^68^Ga- and ^18^F-labelled PSMA tracers, we demonstrated only modest diagnostic accuracy of PSMA PET/CT for local tumour staging, with the pathological tumour stage of the radical prostatectomy specimen serving as the reference standard, yielding AUCs ranging between 0.59 and 0.64 for pT3-stage [5].

The analyses in this previous study were based on visual assessments of PSMA PET/CT scans, as advocated by guidelines [6–8]. However, quantitative parameters obtained from PSMA PET/CT may offer incremental diagnostic value or serve as alternative approaches for evaluating the local extent of primary PCa [9].

The purpose of this study was to explore the value of quantitative PSMA PET/CT parameters in assessing local T-stage, complementing visual assessments, in a large single centre retrospective cohort of patients.

## Materials and methods

### Patients

This study was conducted by the Amsterdam UMC and the Netherlands Cancer Institute – Antoni van Leeuwenhoek (NCI-AVL), both tertiary referral centres within the Prostate Cancer Network the Netherlands. This study is an extension of a retrospective multicentre cohort study of 600 consecutive, predominantly intermediate- and high-risk primary prostate cancer patients, evaluating the value of PSMA PET/CT for local prostate tumour staging [5]. These patients underwent PSMA PET/CT before robot-assisted radical prostatectomy (RARP) according to protocol between 2016 and 2021. To minimize the heterogeneity in PSMA PET/CT data, only patients scanned at NCI-AVL were included in this analysis (*n* = 252). Patients with incomplete imaging data or with PSMA-negative/non-measurable primary prostate tumours were excluded.

### Pre-operative PSMA PET/CT imaging

PSMA PET/CT imaging was performed on EARL-accredited PET systems and in accordance with the EANM guidelines on prostate cancer imaging [10, 11]. The applied PSMA tracers included the ^18^F-labelled tracers: [^18^F]DCFPyL, [^18^F]-JK-PSMA-7, as well as the ^68^Ga-labelled tracer [^68^Ga]Ga-PSMA-11. PET-images were combined with a low-dose CT-scan (120–140 kV, 40–80 mAs with dose modulation) without intravenous contrast enhancement. Additional imaging details have been described previously [5].

### Image analysis

Visual analyses of the PSMA PET/CT scans and assignment of a molecular imaging-based T-stage, i.e. miT-stage (visual), were performed by experienced nuclear medicine physicians as described previously [5]. The probability of extra-capsular extension, ECE (miT3a), was determined using a 3-point Likert scale as 0. not likely (miT2-stage); 1. possible (possible miT3a); and 2. probable (miT3a), whereas the presence of seminal vesicle invasion, SVI (miT3b), was assessed as positive or negative.

**Fig. 1:**
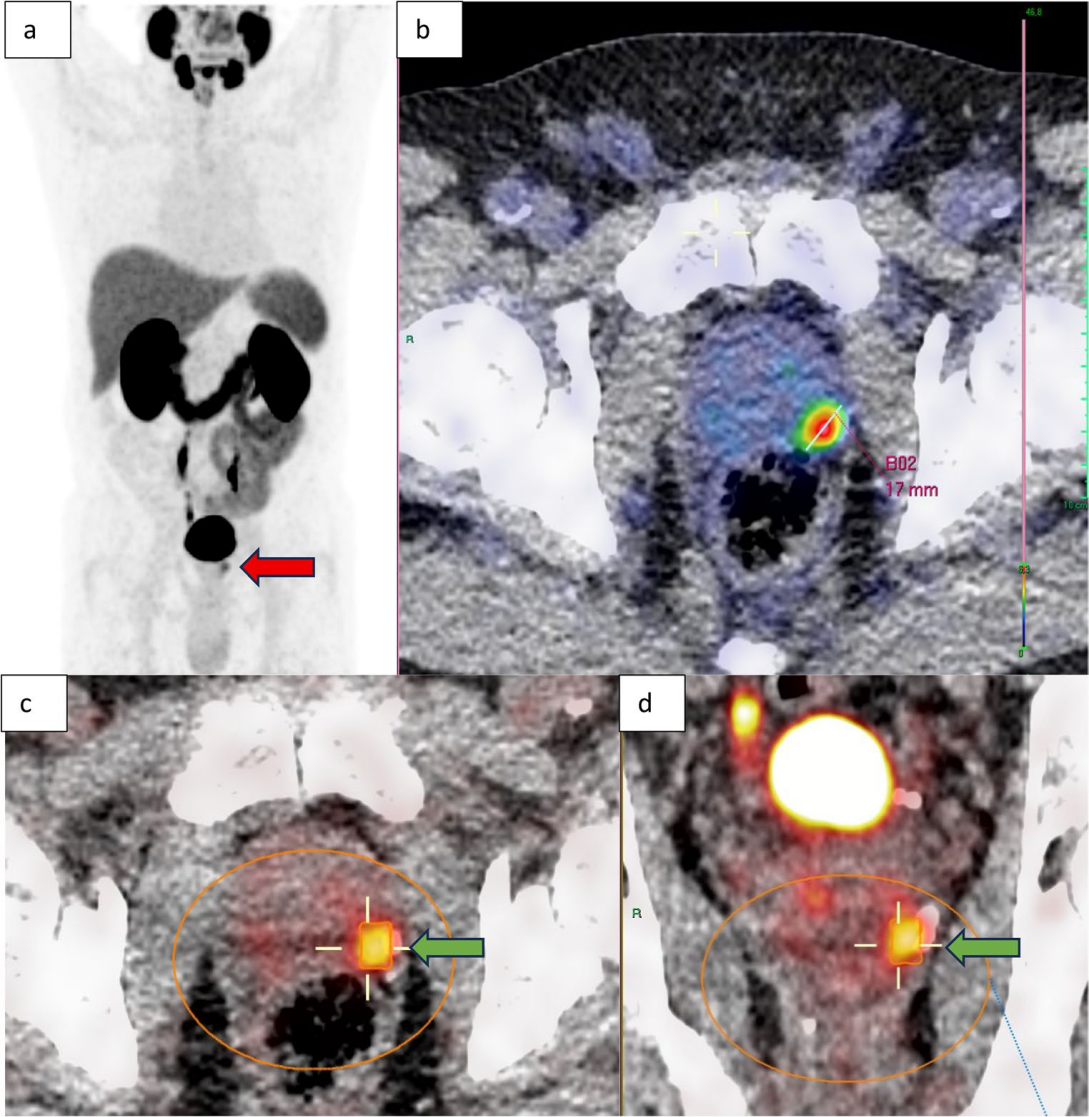
Staging [^18^F]DCPyL PET maximum intensity projection showing focal prostate tumour activity (red arrow) (**a**). Fused axial PET/CT images using a gradual “Rainbow” color look-up table and scaled to tumour SUVmax (in line with methods described by Brauchli et al. and Hvittfeldt et al.) showing LCC measurement on the prostate tumour (PET) – capsule (CT) interface (**b**). Fused axial PET/CT images using standard clinical color look-up table (axial view, c; coronal view, d) showing prostate tumour delineation (green arrow) within a VOI using a SUV threshold of 4, allowing measurement of SUVmax, SUVpeak, PSMA-vol and PSMA-TL

In the current extension study, quantitative parameters of the primary PCa were assessed on the preoperative PSMA PET/CT using a clinical DICOM viewer [12]. On the fused PMSA PET and CT images, a three-dimensional volume of interest (VOI) was drawn over the prostate, encompassing all tumour activity in the prostate and seminal vesicles, while excluding areas with high non-tumourous activity, such as the urinary bladder. Parameters assessed within this VOI were SUVmax and SUVpeak, defined as the maximum standardized uptake value (SUV) and the 1cm3 with the average highest SUV within the VOI, respectively [13]. Furthermore, within this VOI a PSMA PET-based tumour volume (PSMA-vol) was calculated as well as the total lesion PSMA expression (PSMA-TL), defined as PSMA PET-based tumour volume multiplied by the average uptake value. A fixed minimum SUV threshold of 4 was applied, serving as distinction between tumourous and non-tumourous prostate activity [9, 14]. In case of multifocality, all lesions were included, using one or multiple VOIs. Finally, the tumour longest capsule contact (LCC) was determined according to published methods [15, 16], defined as the longest straight line between two points on the tumour (PET) - capsule (CT) interface in the axial, sagittal or coronal orientation, after applying a gradual colour look-up table and appropriate scaling of the PSMA PET (Fig. 1).

Patients with PSMA-negative prostate tumours (PSMA expression score 0/miT0-stage) or with non-measurable tumours due to low activity (SUVmax < 4), diffuse uptake or non-discernability from non-tumour activity, such as diffuse prostatitis or physiologic urine activity, were excluded.

Observers were blinded to all clinical information, including the clinical T-stage, clinical reports of the scans, and results of other imaging.

### Pathological assessment of T-stage after RARP

Histopathology was considered the reference standard for tumour stage (pT-stage) of the primary PCa. All RARP specimens were processed and reported according to the ISUP standard protocols by specialized uropathologists [17] and a pathological T-stage was assigned according to TNM classification (8th ed.) [18].

### Statistical analysis

Statistical analyses were performed using SPSS (IBM; v29). Continuous variables are expressed as mean ± standard deviation (SD), or in case of not normally distributed data as median with interquartile range (IQR). Categorical variables are reported as absolute and relative frequencies. Groupwise comparisons were performed using the Mann-Whitney U test or Pearson’s chi-squared test, as appropriate.

Measures of diagnostic accuracy of visual and quantitative analyses of PSMA PET/CT for predicting pT-stage were calculated including areas under the receiver operating characteristics curves (AUC).

For groupwise comparisons and calculation of diagnostic accuracy, stages miT3b and miT4 were grouped; and overall ≥miT3 was defined as any miT3a, miT3b or miT4. Pathological tumour stage was categorized as pT2, pT3a, and *≥*pT3b. Overall ≥pT3 was defined as any pT3a or ≥pT3b.

Univariable logistic regression analyses were performed to assess diagnostic accuracy of the visual analysis and quantitative parameters separately, combined as well as stratified by tracer (tracer-specific). After identification and removal of strongly colinear variables (Pearson’s *r* > 0.9), multivariable logistic regression analyses were performed using a forward variable selection method (Wald test *p* ≤ 0.05). Thereafter, independent variables were selected to develop risk scores optimized for clinical usability, while retaining diagnostic accuracy. In this, risk categories were determined based on the per variable cut-off value associated with the maximum Youden’s index in the univariable analyses.

All statistical tests were two-tailed, and a value of *p* ≤ 0.05 was considered statistically significant.

## Results

### Patient characteristics

Of 252 included patients, 26 had a PSMA-negative or non-measurable primary prostate tumour, and in 3 patients PET reconstructions according to EARL standards were missing. The remaining 223 evaluable patients had a median (IQR) age of 69 (64–73) years and a median (IQR) initial PSA of 10 ng/ml (7–19). Of these, 133 (59.6%) had a pT3-stage at final pathology. Further clinical and pathological characteristics are listed in Table 1.

**Table 1:** Clinical and histopathological characteristics of 223 evaluable patients who underwent PSMA PET/CT imaging before RARP

		*n =* 223
Age (years), median (IQR)		69 (64–73)
Initial PSA level (ng/ml), median (IQR)		9.6 (7.0–19.0)
Time between PSMA PET/CT and RARP (days), median (IQR)		49 (30–66)
D’Amico Risk Classification;		
Intermediate Risk		109 (48.9%)
High Risk		114 (51.1%)
Pathological Grade Group in prostatectomy specimen;		
ISUP 2		59 (26.5%)
ISUP 3		88 (39.5%)
ISUP 4		32 (14.3%)
ISUP 5		44 (19.7%)
Pathological tumour stage		
pT2		90 (40.4%)
pT3 (overall)		133 (59.6%)
o *pT3a*		• *77 (34.5%)*
o *≥pT3b*		• *56 (25.1%)*

IQR = interquartile range; PSA = prostate-specific antigen; PSMA = prostate-specific membrane antigen; PET = positron emission tomography; CT = computed tomography; RARP = robot-assisted laparoscopic radical prostatectomy; ISUP = International Society of Urological Pathology; p = pathology; T = tumour-stage

### PSMA PET/CT characteristics and results of image analysis

Patients were scanned using three different PSMA tracers: [^68^Ga]Ga-PSMA-11 in 164 (73.5%), [^18^F]DCFPyL in 56 (25.1%), and [^18^F]-JK-PMSA-7 in 3 (1.3%) of the 223 patients. The doses and biodistribution times of the different tracers are provided in Table 2.

**Table 2:** PSMA PET/CT imaging characteristics in 223 evaluable patients

PSMA PET/CT tracer	*n* (%)	Tracer dose (MBq, median (IQR))	Tracer biodistribution time (minutes, median (IQR))
[^68^Ga]Ga-PSMA-11	164 (73.5%)	100 (93–114)	46 (43–50)
[^18^F]DCFPyL	56 (25.1%)	196 (189–203)	60 (57–65)
[^18^F]-JK-PSMA-7	3 (1.3%)	201 (nc)	58 (nc)

MBq = megabecquerel; IQR = interquartile range; nc = non-computable

Results of the PSMA PET/CT-based visual analysis of local tumour (miT-stage) and quantitative parameters are listed in Table 3. Significant differences (*p* < 0.01–0.02) were found in miT-stage, LCC, SUVmax, SUVpeak, PSMA-vol and PSMA-TL between patients with pT2 and pT3-stage primary prostate tumours.

**Table 3:** Comparison of PSMA PET/CT based visual analysis of local tumour stage and quantitative parameters in 223 evaluable patients according to pathological tumour (pT) stage after RARP

	Entire cohort	pT2	≥pT3			*p*-value (pT2 vs. ≥pT3)
			pT3a		≥pT3b	
miT-stage (visual), *n* (%)						
miT2	95 (43%)	51 (23%)	30 (13%)		14 (6%)	0.02*
**≥**miT3						
• Likert 1	62 (28%)	21 (9%)	25 (11%)		16 (7%)	
• Likert 2	66 (30%)	18 (8%)	22 (10%)		26 (12%)	
*of which* ***≥****miT3b*	*17 (8%)*	*0*	*1 (< 1%)*		*16 (7%)*	
LCC mm, median (IQR)	16 (11–22)	13 (9–17)	16 (12–22)		20 (14–33)	< 0.01*
SUVmax median (IQR)	9.0 (6.3–15.7)	7.9 (6.2–12.5)	8.3 (5.6–16.1)		12.0 (8.0–17.1)	0.02*
SUVpeak median (IQR)	6.6 (4.6–10.9)	5.6 (4.2–8.5)	6.3 (4.6–11.6)		8.9 (6.5–13.5)	< 0.01*
PSMA-vol, cm^3^, median (IQR)	4.2 (1.9–9.9)	3.1 (1.2–6.2)	4.2 (1.9–8.0)		8.9 (6.5–13.5)	< 0.01*
PSMA-TL median (IQR)	25.1 (8.9–60.2)	16.0 (6.1–36.4)	27.4 (8.7–52.3)		58.2 (22.9–172.5)	< 0.01*

mi = molecular imaging; LCC = longest capsule contact; SUV = standardised uptake value; PSMA-vol = PSMA PET basedtumour volume; PSMA-TL = total lesion PSMA expression; *denotes significance at alpha .05

### Diagnostic accuracy of PSMA PET/CT*-*based visual analysis and quantitative parameters for pathological T-staging

Univariable logistic regression analyses of visual assessment yielded AUCs of 0.59, 0.64, and 0.63 for predicting pT3a, ≥pT3b, and overall ≥ pT3-stage, respectively. Quantitative parameters showed AUCs of 0.53–0.63 for prediction of pT3a, 0.64–0.74 for ≥ pT3b, and 0.59–0.69 for ≥ pT3-stage. All visual analyses and quantitative parameters were statistically significant predictors of pT-stage (*p* < 0.01–0.04), except for SUVmax and SUVpeak in predicting pT3a-stage (Table 4).

**Table 4:** Univariable logistic regression analyses. Diagnostic accuracy of PSMA PET/CT based visual analysis of local tumour stage (miT-stage) and quantitative parameters for pathological tumour stage (pT-stage) in 223 evaluable patients, regarding **A**. pT3a-stage; **B**. ≥pT3b-stage and **C**. ≥pT3-stage

	AUC [95% CI]	max. Youden’s index	Cut-off value	sensitivity	specificity	*p*-value
**A. pT3a-stage**						
miT3a-stage (visual; Likert)	0.59 **[**0.50–0.68]	0.18	≥2	0.61	0.57	0.04*
LCC (mm)	0.63 **[**0.55–0.72]	0.21	≥17	0.49	0.71	< 0.01*
SUVmax	0.53 **[**0.44–0.62]	0.13	12.8	0.36	0.77	0.57
SUVpeak	0.57 [0.48–0.65]	0.15	9.5	0.36	0.79	0.15
PSMA-vol (cm^3^)	0.60 **[**0.51–0.68]	0.17	3.5	0.58	0.59	0.03*
PSMA-TL	0.59 [0.50–0.67]	0.19	28.6	0.49	0.70	0.05*
**B. ≥pT3b-stage**						
miT3b-stage (visual)	0.64 **[**0.55–0.73]	n.a.^†^	n.a.^†^	0.29	0.99	< 0.01*
LCC (mm)	0.71 **[**0.63–0.79]	0.35	≥18	0.64	0.71	< 0.01*
SUVmax	0.66 **[**0.58–0.73]	0.27	9.9	0.66	0.61	< 0.01*
SUVpeak	0.68 **[**0.60–0.75]	0.34	6.4	0.79	0.56	< 0.01*
PSMA-vol (cm^3^)	0.74 **[**0.66–0.82]	0.40	6.9	0.64	0.75	< 0.01*
PSMA-TL	0.74 **[**0.66–0.81]	0.39	43.9	0.63	0.76	< 0.01*
**C. ≥pT3-stage**						
miT3-stage (visual; Likert)	0.63 **[**0.56–0.70]	0.24	≥2	0.67	0.57	< 0.01*
LCC (mm)	0.69 **[**0.62–0.76]	0.30	17.5	0.50	0.80	< 0.01*
SUVmax	0.59 **[**0.51–0.67]	0.17	11.6	0.45	0.72	0.02*
SUVpeak	0.63 **[**0.55–0.70]	0.25	6.2	0.65	0.60	< 0.01*
PSMA-vol (cm^3^)	0.67 **[**0.59–0.73]	0.27	3.3	0.70	0.57	< 0.01*
PSMA-TL	0.63 **[**0.56–0.70]	0.28	28.6	0.57	0.71	< 0.01*

AUC = area under the receiver operator curve; CI = confidence interval; ^†^n.a. not applicable (binary value); *denotes significance at alpha 0.05

When stratified by tracer type, diagnostic accuracy rates varied substantially for certain visual and quantitative analyses; however, none of these differences reached statistical significance (Table 5).

**Table 5:** Comparison of tracer-specific* diagnostic accuracy of PSMA PET/CT based visual analysis of local tumour stage (miT-stage) and quantitative parameters for pathological tumour stage (pT-stage) in 223 evaluable patients, regarding **A**. pT3a-stage; **B**. ≥pT3b-stage and **C**. ≥pT3-stage

	AUC [^68^Ga]Ga-PSMA-11	AUC [^18^F]DCFPyL		AUC difference	*p*-value
**A. pT3a-stage**					
miT3a-stage (visual)	0.58		0.57	0.01	0.89
LCC	0.60		0.71	0.12	0.21
SUVmax	0.57		0.38	0.18	0.08
SUVpeak	0.59		0.46	0.14	0.20
PSMA-vol	0.58		0.59	< 0.01	0.97
PSMA-TL	0.58		0.56	0.02	0.83
**B. ≥pT3b-stage**					
miT3b-stage (visual)	0.63		0.71	0.08	0.46
LCC	0.68		0.80	0.12	0.20
SUVmax	0.64		0.73	0.09	0.33
SUVpeak	0.67		0.73	0.07	0.44
PSMA-vol	0.72		0.81	0.09	0.29
PSMA-TL	0.72		0.80	0.08	0.38
**C. ≥pT3-stage**					
miT3-stage (visual)	0.61		0.64	0.02	0.79
LCC	0.65		0.77	0.12	0.12
SUVmax	0.61		0.50	0.12	0.19
SUVpeak	0.64		0.56	0.09	0.33
PSMA-vol	0.66		0.68	0.03	0.75
PSMA-TL	0.66		0.65	< 0.01	0.98

*No tracer-specific values given for [^18^F]-JK-PSMA-7 due to low number of cases (*n* = 3)

Strong collinearity was observed between SUVmax and SUVpeak, and between PSMA-vol and PSMA-TL (Pearson’s *r* > 0.98 and *r* > 0.96, respectively). SUVpeak was selected over SUVmax due to its superior diagnostic accuracy, whereas PSMA-vol was preferred over PSMA-TL for greater practical applicability.

Multivariable logistic regression analyses using all remaining variables showed that LCC was the only independent predictor for pT3a-stage, with a model AUC of 0.63 (95% CI 0.55–0.72). Independent predictors for ≥pT3b-stage were visual miT3b-stage, LCC and PSMA-vol with a model AUC of 0.79 (95% CI 0.72–0.87). For overall ≥pT3-stage, LCC and PSMA-vol were identified as independent predictors, with a model AUC of 0.70 (95% CI 0.64–0.77) (Table 6).

**Table 6:** Independent predictors from multivariable logistic regression analysis of PSMA PET/CT based visual analysis of local tumour stage (miT-stage) and quantitative parameters for pathological tumour stage (pT-stage) in 223 evaluable patients, regarding **A**. pT3a-stage; **B**. ≥pT3b-stage and **C**. ≥pT3-stage

	B	SE	Odds ratio [95% CI]	*p*-value	Model AUC [95% CI]
**A. pT3a-stage**					
LCC	0.055	0.020	1.057 [1.015–1.100]	< 0.01*	
					0.63 [0.55–0.72]^†^
**B. ≥pT3b-stage**					
miT3b-stage (visual)	3.770	1.072	43.389 [5.308–354.642]	< 0.01*	
LCC	0.047	0.022	1.048 [1.003–1.095]	< 0.01*	
PSMA-vol	0.058	0.026	1.060 [1.007–1.116]	< 0.01*	
					0.79 [0.72–0.87]
**C. ≥pT3-stage**					
LCC	0.056	0.021	1.058 [1.015–1.102]	< 0.01*	
PSMA-vol	0.054	0.027	1.056 [1.000–1.114]	0.05*	
					0.70 [0.64–0.77]

B = b-coefficient, SE = standard error for b-coefficient, CI = confidence interval, *denotes significance at alpha 0.05; ^†^model consists of a single independent predictor

Clinically usable risk scores were developed based on the results of the multivariable logistic regression analysis (Table 7).

**Table 7:** Diagnostic accuracy of multivariable analysis-based clinical risk scores of PSMA PET/CT based miT-stage and quantitative parameters for **A**. pT3a-stage; **B**. ≥pT3b-stage and **C**. ≥pT3-stage

**A. pT3a-stage**											
**LCC (mm)**		**no pT3a;** ***n***	**pT3a;** ***n***	**Total;** ***n***		**Cut-off**	**Sensitivity [95% CI]**	**Specificity** **[95% CI]**	**PPV** **[95% CI]**	**NPV** **[95% CI]**	
**0–11**		33	17	50							
**12–17**		39	29	68		≥12	0.78 [0.68–0.86]	0.37 [0.27–0.47]	0.51 [0.42–0.60]	0.66 [0.52–0.78]	
**18–23**		10	16	26		≥18	0.40 [0.30–0.51]	0.80 [0.71–0.87]	0.63 [0.49–0.76]	0.61 [0.52–0.70]	
**≥24**		8	15	23		≥24	0.20 [0.12–0.29]	0.91 [0.84–0.96]	0.65 [0.45–0.82]	0.57 [0.49–0.65]	
**Total;** ***n***		90	77	167			**AUC** 0.62 [95% CI 0.54–0.71]				
**B. ≥pT3b-stage**											
**Risk category**		**< pT3b;** ***n***	**≥pT3b;** ***n***	**Total;** ***n***		**Cut-off**	**Sensitivity** **[95% CI]**	**Specificity** **[95% CI]**	**PPV** **[95% CI]**	**NPV** **[95% CI]**	
**LR**		67	5	72							
**L-IR**		66	12	78		≥L-IR	0.91 [0.82–0.97]	0.40 [0.33–0.48]	0.34 [0.27–0.42]	0.93 [0.86–0.98]	
**H-IR**		33	30	63		≥H-IR	0.70 [0.73–0.85]	0.80 [0.73–0.85]	0.53 [0.42–0.65]	0.89 [0.83–0.93]	
**HR**		1	9	10		≥HR	0.16 [0.08–0.27]	0.99 [0.97–1.00]	0.90 [0.63–0.99]	0.74 [0.67–0.80]	
**Total;** ***n***		167	56	223			**AUC** 0.79 [95% CI 0.72–0.86]				
	Risk categories for ≥pT3b. LR, L-IR-, H-IR or HR in case of 0, 1, 2, or 3 positive criteria, respectively: miT3b-stage (visual), LCC ≥18 mm, PSMA-vol ≥7cm^3^										
**C. ≥pT3-stage**											
**Risk category**		**pT2;** ***n***	**≥pT3;** ***n***	**Total;** ***n***		**Cut-off**	**Sensitivity** **[95% CI]**	**Specificity** **[95% CI]**	**PPV** **[95% CI]**	**NPV** **[95% CI]**	
**LR**		45	27	72							
**IR**		33	52	85		≥IR	0.80 [0.72–0.86]	0.50 [0.40–0.60]	0.70 [0.63–0.77]	0.63 [0.51–0.73]	
**HR**		12	54	66		≥HR	0.41 [0.32–0.49]	0.87 [0.79–0.93]	0.82 [0.71–0.90]	0.50 [0.42–0.58]	
**Total;** ***n***		90	133	223			**AUC** 0.70 [95% CI 0.63–0.77]				
	Risk categories for ≥pT3. LR, IR-, or HR in case of 0, 1, or 2 positive criteria, respectively: LCC ≥18 mm, PSMA-vol ≥3.3cm^3^										
	LR = low risk; IR = intermediate risk; L-IR = low-intermediate risk; H-IR = high-intermediate risk; HR = high risk; Risk calculated as *n* of pathological T3a/≥T3b/≥T3 cases divided per total *n* of cases within the specified risk category.										

For pT3a-stage, three LCC cut-off values with a fixed 6 mm increment (12, 18, and 24 mm) yielded sensitivities and specificities of 0.78 and 0.37, 0.40 and 0.80, and 0.20 and 0.91, respectively, with an overall AUC of 0.62 (95% CI, 0.54–0.71).

For ≥pT3b-stage, four risk categories (low, low-intermediate, high-intermediate, and high risk) were defined using three cut-off levels based on the presence of zero, one, two or three of the following criteria: miT3b-stage (visual), LCC ≥18 mm, and PSMA-vol ≥7cm3. These cut-off levels yielded sensitivities and specificities of 0.91 and 0.40, 0.70 and 0.80, and 0.16 and 0.99, respectively, with a model AUC of 0.79 (95% CI 0.72–0.86).

For overall ≥pT3-stage, three risk categories (low, intermediate, and high risk) were defined with two cut-off levels based on the presence of zero, one or two of the following criteria: LCC ≥18 mm and PSMA-vol ≥3.3cm3. These cut-off levels yielded sensitivities and specificities of 0.80 and 0.50, and 0.41 and 0.87, respectively, with a model AUC of 0.70 (95% CI 0.63–0.77).

**Fig. 2:**
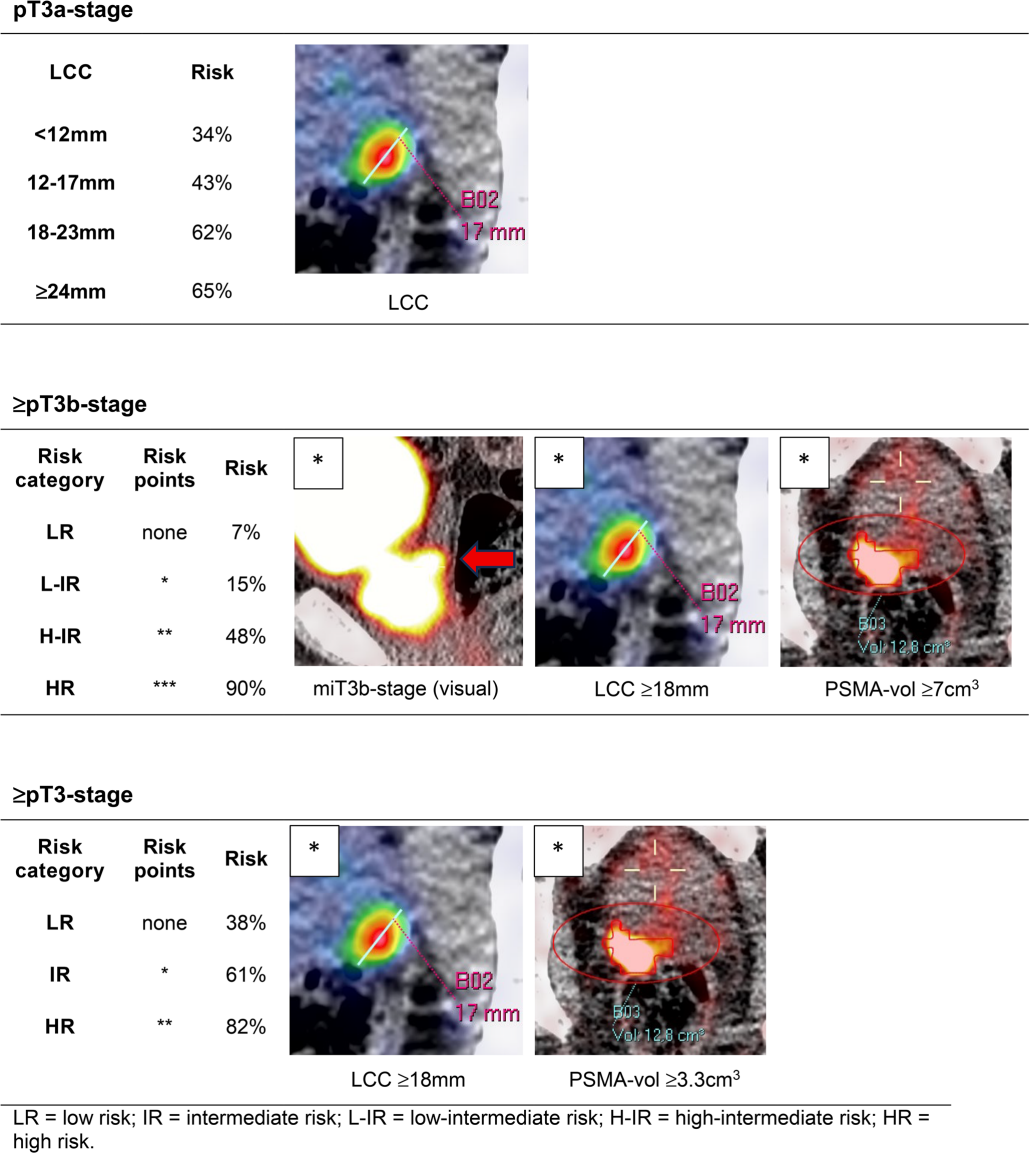
Pictorial overview of image criteria, risk categories and associated risk percentages for extraprostatic extension

A pictorial overview of image criteria, risk categories and associated risk percentages for extraprostatic extension is shown in Fig. 2.

## Discussion

PSMA PET/CT has shown potential in the local (T) staging of primary PCa, where the identification of T3 disease holds prognostic and therapeutic significance. Initial smaller studies showed promising diagnostic accuracy values for PSMA PET/CT-based detection of T3 disease [19]. However, subsequent large-scale studies demonstrated only modest diagnostic utility, reporting AUC-values between 0.59 and 0.64 for pathological (p)T3-stage disease [5, 20]. These studies relied on visual assessments of PSMA PET/CT scans, as advocated by the PROMISE and E-PSMA guidelines [6, 7, 11]. In addition to visual assessments, quantitative PSMA PET/CT parameters, such as SUVmax and tumour volume, may provide complementary diagnostic information. The purpose of this study was to explore the value of quantitative PSMA PET/CT parameters in addition to the visual assessment of the local T-stage.

The present study is an extension of an earlier study from our department in which the diagnostic accuracy and observer variability of PSMA PET/CT for the local staging of primary PCa was investigated [5]. The diagnostic accuracy of the visual analyses in the current cohort (AUCs of 0.59, 0.64 and 0.63 for pT3a, ≥pT3b and (overall) ≥ pT3-stage, respectively) aligns closely with that of our previous publication (AUCs of 0.59, 0.64 and 0.64 for pT3a, ≥pT3b and ≥ pT3-stage, respectively). Data from the present study showed that on univariable analyses, single quantitative parameters surpass the diagnostic accuracy of visual analyses with reported AUCs of up to 0.63 for pT3a, 0.74 for ≥ pT3b and 0.69 for ≥ pT3-stage. Multivariable analyses identified LCC as the single independent predictor for pT3a stage, miT-stage, LCC and PSMA-vol as independent predictors for ≥ pT3b-stage; and LCC and PSMA-vol as independent predictors for ≥ pT3-stage. Clinical risk models incorporating these predictors yielded AUCs of 0.62 for pT3a, 0.79 for ≥ pT3b and 0.70 for ≥ pT3-stage, substantially exceeding the diagnostic accuracy of visual analyses alone. No statistically significant differences in diagnostic accuracy were found between PSMA PET tracers [^68^Ga]Ga-PSMA-11 and [^18^F]DCFPyL, consistent with the earlier multicentre study using visual analyses alone [5]. However, several individual quantitative parameters showed substantial differences between PSMA PET tracers; it cannot be excluded that a larger number of patients per tracer cohort could have led to significant differences.

Among quantitative PET parameters, SUVmax is likely the most commonly used in clinical practice. It has been shown in previous studies that the SUVmax of the primary PCa on PSMA PET/CT is related to pathological T-stage [20–23]. However, its discriminative power for detecting EPE is generally limited, and its robustness may be limited by its variability dependent on tracer type, PET system and used protocols [22]. Other quantitative parameters have been less extensively studied. The LCC, a well-known metric in prostate MRI [24], has been evaluated in only a few PSMA PET/CT studies. High diagnostic accuracy values for detecting ECE were reported by Brauchli et al. (74% sensitivity, 86% specificity), Hvittfeldt et al. (46% sensitivity, 91% specificity, AUC 0.70) and Woo et al. (67% sensitivity, 81% specificity AUC 0.80), using LCC cut-off values of 10 mm, 14 mm, and 15 mm respectively [15, 16, 23]. Laudicella et al. demonstrated that PSMA PET-based tumour volume using a SUV threshold > 4 (i.e., PSMA-vol) was related to T-stage, yielding an AUC of 0.709 for detecting (overall) T3-stage based on a cut-off of 4.41 cm^3^. They reported similar results for the related parameter PSMA-total lesion [9]. Woo et al. reported a sensitivity of 82%, specificity of 69% and AUC of 0.80 for extracapsular extension, using a single-dimensional PSMA PET/CT-based tumour size with a cut-off of 1.2 cm [23].

To the best of our knowledge, this is the first study to present the value of quantitative PSMA PET/CT parameters combined with visual analyses for the assessment of the local extent of disease, using multivariable analyses and providing clinically usable risk categories with variable cut-off levels. The proposed quantitative (T3a-stage) or combined visual and quantitative assessment methods (≥ T3b- and overall ≥ T3-stage) require one or multiple measurements, taking short time to perform. These measurements are easily performed by trained nuclear medicine physicians, whereas reproducibility of these quantitative assessments have been reported to be substantial for LCC [16] and nearly perfect for PSMA-vol [9].

The added value of quantitative assessments compared to visual assessments has been clearly demonstrated in our data, particularly for the assessment of ≥ pT3b-stage. Our results apparently differ from those from a recent large multicentre study, in which quantitative PSMA PET assessments did not show additional value compared to visual analyses and established risk factors [25]. However, this study of Sweere et al. differs in outcome measure (BCR free survival) and methodology (prognostic, longitudinal study), as well as the incorporation of MRI and other non-imaging risk factors in the multivariable analyses, which influences the weight and significance of the individual variables. Furthermore, the diagnostic accuracy of the visual analyses in our study was rather low as has been discussed previously [5], leaving room for improvement of diagnostic accuracy by using quantitative assessments. Still, for pT3a-stage (ECE) diagnostic accuracy using quantitative assessments was lower than reported in earlier studies [9, 15, 16, 23]. This discrepancy may be attributed to differences in study population, study design, and technical factors, among others. Furthermore, quantitative assessments in the present study may have been hampered by the use of (initially) rather low doses of the [^68^Ga]Ga-PSMA-11 tracer and the use of older generation PET-scanners, leading to lower spatial resolution and, as a consequence, larger partial volume effects, and increased noise compared to current digital and large axial field-of-view PET systems.

Specifically, PSMA PET/CT-based LCC measurement may have been suboptimal due to linear measurement in a single plane, whereas a curvilinear measurement may better represent the true tumour-capsule contact. Though, differences in LCC measurement arise mainly in the larger prostate tumours, which already fall within the high-risk category, therefore the impact of different LCC measuring methods may be limited. Moreover, the optimal method of LCC measurement (straight versus curvilinear) remains a subject of debate in MRI as well [24]. Secondly, the use of lowdose, non-contrast-enhanced CT in current cohort may have been suboptimal, as the prostate capsule has to be clearly discerned for measuring the tumour-capsule interface. Thirdly, adequate LCC measurement may have been hampered by misalignment between PET and CT, for instance due to urinary bladder filling during the image acquisition. Nonetheless, LCC proved a valuable quantitative parameter both in univariable and multivariable analyses. Interestingly, although LCC is intended for prediction of ECE, it also emerged as an independent predictor for ≥ pT3b-stage in multivariable analysis, whereas SUVpeak and SUVmax did not. This cannot be solely attributed to a volume effect, as PSMA-vol showed to be a separate, independent predictor for pT3b-stage. Possibly, tumours with high risk of ECE have a high probability of SVI as well. In our study, this specific correlation could not be tested as only the highest T-stage was recorded, without separate documentation of ECE and SVI.

It should be noted that 10,3% of patients were not suitable for quantitative assessment of the primary tumour using PSMA PET/CT, which is higher than the non-evaluable proportion of patients in the parent cohort due to PSMA-negative primary tumour (3.5%) [5]. In these additional non-evaluable cases, quantification of the tumour was not possible due to non-discernability from prostatitis or physiological activity. Still, in such cases, PSMA PET/CT-based T-stage assessment remains feasible using visual analyses alone.

Prostate MRI has been established as a pivotal and highly accurate diagnostic instrument to determine local tumour stage [4]. A previous, large meta-analysis showed that MRI generally has high specificity (82–88%) for detection of T3-disease, while sensitivity is limited (51–57%) and heterogeneous among studies [26]. The current study shows that a combined visual and quantitative PSMA PET/CT assessment yields sufficient (T3a-stage) to good (T3b- and overall T3-stage) diagnostic accuracy. A recent multicentre study including 550 patients reported reasonable diagnostic accuracies for single-modality prostate MRI and PSMA PET/CT, yielding AUCs of 0.69 and 0.62 for ECE, respectively, and 0.66 and 0.70 for SVI, respectively. However, combining the highest T-stage from both modalities significantly improved diagnostic accuracy, particularly for SVI detection (AUC 0.76) [27]. The potential of combining quantitative PSMA PET/CT assessments with prostate MRI, and strategies for resolving discrepancies between the two modalities may be subject of further research.

Strengths of the current study include the number of included patients and the comparison with histopathology as reference standard. Additionally, patients were included in a high-volume, dedicated multicentre prostate cancer network with a prospectively maintained database. Importantly, beyond reporting univariable associations, a comprehensive multivariable analysis was performed, leading to clinically usable risk scores that are directly applicable in clinical practice. Through variable cut-off levels, the models allow for a more sensitive or specific approach, tailored to clinical needs and the prevalence of T3 disease in the underlying population.

A limitation of current study is the retrospective design, which may have introduced bias. Furthermore, quantitative assessments were performed by a single observer. Although robustness seems high [9, 16], current study provides no data on observer agreement. Also, while quantitative parameters are seemingly objective, findings of current study may not be fully generalizable to other patient populations, other PET systems and protocols. The assessments and analyses were based on a per-patient analysis, which may have been less precise than a per-lesion analysis in case of multifocal tumours. Finally, quantitative assessments depend on PSMA expression and the results may not be applicable to low or low-intermediate risk PCa, which generally exhibits lower PSMA expression.

## Conclusion

In a large, single-center cohort study of 223 evaluable patients with newly diagnosed intermediate- and high-risk prostate cancer, we demonstrated that quantitative parameters derived from PSMA PET/CT provide good additional diagnostic accuracy for detecting extraprostatic tumor extension, particularly for ≥ pT3b-stage disease, outperforming visual analysis (miT-stage) alone.

## Data Availability

The datasets generated and analysed during the current study are not publicly available as these contain individual person’s data but are available from the corresponding author on reasonable request, after pseudonymization of the data and legal agreement.
