# Correction to: Addition of quantitative imaging parameters to visual analysis improves the accuracy of PSMA PET/CT for the local staging of primary prostate cancer

**DOI:** 10.1007/s00259-026-07861-y

**Published:** 2026-03-25

**Authors:** Maarten L. Donswijk, Rosemarijn H. Ettema, Maurits Wondergem, Zing Cheung, Henk G. van der Poel, Daniela E. Oprea-Lager, André N. Vis

**Affiliations:** 1 Department of Nuclear Medicine, The Netherlands Cancer Institute - Antoni van Leeuwenhoek, Amsterdam, The Netherlands; 2 Department of Urology, Amsterdam University Medical Centres Amsterdam, Amsterdam, The Netherlands; 3 Prostate cancer network, Amsterdam, The Netherlands; 4 Department of Urology, St. Antonius Hospital, Nieuwegein, The Netherlands; 5 Department of Radiology and Nuclear Medicine, University Medical Centre Utrecht, Utrecht, The Netherlands; 6 Department of Nuclear Medicine, Noordwest Ziekenhuisgroep, Alkmaar, The Netherlands; 7 Department of Urology, The Netherlands Cancer Institute - Antoni van Leeuwenhoek, Amsterdam, The Netherlands; 8 Department of Medical Imaging, Radboud University Medical Center, Nijmegen, The Netherlands

**Correction to: European Journal of Nuclear Medicine and Molecular Imaging**

10.1007/s00259-025-07757-3

The authors regret that the version of Tables 3, 6 and 7 that appeared in the original published article are incorrect.

The correct and incorrect version of Tables 3, 6 and 7 are shown below.

Below is the incorrect Table 3.

**Table 3.** Comparison of PSMA PET/CT based visual analysis of local tumour stage and quantitative parameters in 223 evaluable patients according to pathological tumour (pT) stage after RARP

**Table:** 

	Entire cohort	pT2	≥pT3			*p*-value (pT2 vs. ≥pT3)
			pT3a		³pT3b	
**miT-stage (visual)**, *n* (%)						
miT2	95 (43%)	51 (23%)	30 (13%)		14 (6%)	0.02*
≥miT3						
• Likert 1	62 (28%)	21 (9%)	25 (11%)		16 (7%)	
• Likert 2	66 (30%)	18 (8%)	22 (10%)		26 (12%)	
*of which ≥miT3b*	*17 (8%)*	*0*	*1 (< 1%)*		*16 (7%)*	
**LCC** mm, median (IQR)	16 (11–22)	13 (9–17)	16 (12–22)		20 (14–33)	< 0.01*
**SUVmax** median (IQR)	9.0 (6.3–15.7)	7.9 (6.2–12.5)	8.3 (5.6–16.1)		12.0 (8.0-17.1.0.1)	0.02*
**SUVpeak** median (IQR)	6.6 (4.6–10.9)	5.6 (4.2–8.5)	6.3 (4.6–11.6)		8.9 (6.5–13.5)	< 0.01*
**PSMA-vol**, cm^3^, median (IQR)	4.2 (1.9–9.9)	3.1 (1.2–6.2)	4.2 (1.9-8.0.9.0)		8.9 (6.5–13.5)	< 0.01*
**PSMA-TL** median (IQR)	25.1 (8.9–60.2)	16.0 (6.1–36.4)	27.4 (8.7–52.3)		58.2 (22.9-172.5.9.5)	< 0.01*

mi = molecular imaging; LCC = longest capsule contact; SUV = standardised uptake value; PSMA-vol = PSMA PET based tumour volume; PSMA-TL = total lesion PSMA expression; *denotes significance at alpha .05

Below is the correct Table 3.

**Table 3.** Comparison of PSMA PET/CT based visual analysis of local tumour stage and quantitative parameters in 223 evaluable patients according to pathological tumour (pT) stage after RARP

**Table:** 

	Entire cohort	pT2	≥pT3			*p*-value (pT2 vs. ≥pT3)
			pT3a		≥pT3b	
**miT-stage (visual)**, *n* (%)						
miT2	95 (43%)	51 (23%)	30 (13%)		14 (6%)	0.02*
≥miT3						
• Likert 1	62 (28%)	21 (9%)	25 (11%)		16 (7%)	
• Likert 2	66 (30%)	18 (8%)	22 (10%)		26 (12%)	
*of which ≥miT3b*	*17 (8%)*	*0*	*1 (< 1%)*		*16 (7%)*	
**LCC** mm, median (IQR)	16 (11–22)	13 (9–17)	16 (12–22)		20 (14–33)	< 0.01*
**SUVmax** median (IQR)	9.0 (6.3–15.7)	7.9 (6.2–12.5)	8.3 (5.6–16.1)		12.0 (8.0-17.1)	0.02*
**SUVpeak** median (IQR)	6.6 (4.6–10.9)	5.6 (4.2–8.5)	6.3 (4.6–11.6)		8.9 (6.5–13.5)	< 0.01*
**PSMA-vol**, cm^3^, median (IQR)	4.2 (1.9–9.9)	3.1 (1.2–6.2)	4.2 (1.9-8.0)		8.9 (6.5–13.5)	< 0.01*
**PSMA-TL** median (IQR)	25.1 (8.9–60.2)	16.0 (6.1–36.4)	27.4 (8.7–52.3)		58.2 (22.9-172.5)	< 0.01*

mi = molecular imaging; LCC = longest capsule contact; SUV = standardised uptake value; PSMA-vol = PSMA PET based tumour volume; PSMA-TL = total lesion PSMA expression; *denotes significance at alpha .05

Below is the incorrect Table 6.

**Table 6.** Independent predictors from multivariable logistic regression analysis of PSMA PET/CT based visual analysis of local tumour stage (miT-stage) and quantitative parameters for pathological tumour stage (pT-stage) in 223 evaluable patients, regarding **A**. pT3a-stage; **B**. ≥pT3b-stage and **C**. ≥pT3-stage

**Table:** 

	B	SE	Odds ratio [95% CI]	*p*-value	Model AUC [95% CI]
**A**. **pT3a-stage**					
LCC	0.055	0.020	1.057 [1.015-1.100.015.100]	< 0.01*	
					0.63 [0.55–0.72]^†^
**B. ≥pT3b-stage**					
miT3b-stage (visual)	3.770	1.072	43.389 [5.308-354.642.308.642]	< 0.01*	
LCC	0.047	0.022	1.048 [1.003–1.095]	< 0.01*	
PSMA-vol	0.058	0.026	1.060 [1.007–1.116]	< 0.01*	
					0.79 [0.72–0.87]
**C. ≥pT3-stage**					
LCC	0.056	0.021	1.058 [1.015–1.102]	< 0.01*	
PSMA-vol	0.054	0.027	1.056 [1.000-1.114.000.114]	0.05*	
					0.70 [0.64–0.77]

B = b-coefficient, SE= standard error for b-coefficient, CI= confidence interval, *denotes significance at alpha .05; ^†^model consists of a single independent predictor

Below is the correct Table 6.

**Table 6.** Independent predictors from multivariable logistic regression analysis of PSMA PET/CT based visual analysis of local tumour stage (miT-stage) and quantitative parameters for pathological tumour stage (pT-stage) in 223 evaluable patients, regarding A. pT3a-stage; B. ≥pT3b-stage and C. ≥pT3-stage

**Table:** 

	B	SE	Odds ratio [95% CI]	*p*-value	Model AUC [95% CI]
**A**. **pT3a-stage**					
LCC	0.055	0.020	1.057 [1.015-1.100]	< 0.01*	
					0.63 [0.55–0.72]^†^
**B. ≥pT3b-stage**					
miT3b-stage (visual)	3.770	1.072	43.389 [5.308-354.642]	< 0.01*	
LCC	0.047	0.022	1.048 [1.003–1.095]	< 0.01*	
PSMA-vol	0.058	0.026	1.060 [1.007–1.116]	< 0.01*	
					0.79 [0.72–0.87]
**C. ≥pT3-stage**					
LCC	0.056	0.021	1.058 [1.015–1.102]	< 0.01*	
PSMA-vol	0.054	0.027	1.056 [1.000-1.114]	0.05*	
					0.70 [0.64–0.77]

B = b-coefficient, SE= standard error for b-coefficient, CI= confidence interval, *denotes significance at alpha .05; ^†^model consists of a single independent predictor

Below is the incorrect Table 7.

**Table 7.** Diagnostic accuracy of multivariable analysis-based clinical risk scores of PSMA PET/CT based miT-stage and quantitative parameters for **A**. pT3a-stage; **B**. ≥pT3b-stage and **C**. ≥pT3-stage

**Table:** 

A. pT3a-stage								
**LCC (mm)**	**no pT3a;** ***n***	**pT3a;** ***n***	**Total;** ***n***	**Cut-off**	**Sensitivity [95% CI]**	**Specificity** **[95% CI]**	**PPV** **[95% CI]**	**NPV** **[95% CI]**
**0–11**	33	17	50					
**12–17**	39	29	68	≥12	0.78 [0.68–0.86]	0.37 [0.27–0.47]	0.51 [0.42–0.60]	0.66 [0.52–0.78]
**18–23**	10	16	26	≥18	0.40 [0.30–0.51]	0.80 [0.71–0.87]	0.63 [0.49–0.76]	0.61 [0.52–0.70]
**≥24**	8	15	23	≥24	0.20 [0.12–0.29]	0.91 [0.84–0.96]	0.65 [0.45–0.82]	0.57 [0.49–0.65]
**Total**; ***n***	90	77	167		**AUC** 0.62 [95% CI 0.54–0.71]			
**B. ≥pT3b-stage**								
**Risk category**	**<pT3b;** ***n***	**≥pT3b;** ***n***	**Total;** ***n***	**Cut-off**	**Sensitivity** **[95% CI]**	**Specificity** **[95% CI]**	**PPV** **[95% CI]**	**NPV** **[95% CI]**
**LR**	67	5	72					
**L-IR**	66	12	78	≥L-IR	0.91 [0.82–0.97]	0.40 [0.33–0.48]	0.34 [0.27–0.42]	0.93 [0.86–0.98]
**H-IR**	33	30	63	≥H-IR	0.70 [0.73–0.85]	0.80 [0.73–0.85]	0.53 [0.42–0.65]	0.89 [0.83–0.93]
**HR**	1	9	10	≥HR	0.16 [0.08–0.27]	0.99 [0.97-1.00.97.00]	0.90 [0.63–0.99]	0.74 [0.67–0.80]
**Total;** ***n***	167	56	223		**AUC** 0.79 [95% CI 0.72–0.86]			
Risk categories for ≥pT3b. LR, L-IR-, H-IR or HR in case of 0, 1, 2, or 3 positive criteria, respectively: miT3b-stage (visual), LCC ≥18mm, PSMA-vol ≥7cm^3^								
**C. ≥pT3-stage**								
**Risk category**	**pT2;** ***n***	**≥pT3;** ***n***	**Total;** ***n***	**Cut-off**	**Sensitivity** **[95% CI]**	**Specificity** **[95% CI]**	**PPV** **[95% CI]**	**NPV** **[95% CI]**
**LR**	45	27	72					
**IR**	33	52	85	≥IR	0.80 [0.72–0.86]	0.50 [0.40–0.60]	0.70 [0.63–0.77]	0.63 [0.51–0.73]
**HR**	12	54	66	≥HR	0.41 [0.32–0.49]	0.87 [0.79–0.93]	0.82 [0.71–0.90]	0.50 [0.42–0.58]
**Total;** ***n***	90	133	223		**AUC** 0.70 [95% CI 0.63–0.77]			
Risk categories for ≥pT3. LR, IR-, or HR in case of 0, 1, or 2 positive criteria, respectively: LCC ≥18mm, PSMA-vol ≥3.3cm^3^								

LR = low risk; IR = intermediate risk; L-IR = low-intermediate risk; H-IR = high-intermediate risk; HR = high risk; Risk calculated as *n* of pathological T3a/≥T3b/≥T3 cases divided per total *n* of cases within the specified risk category.

Below is the correct Table 7.

**Table 7.** Diagnostic accuracy of multivariable analysis-based clinical risk scores of PSMA PET/CT based miT-stage and quantitative parameters for **A**. pT3a-stage; **B**. ≥pT3b-stage and **C**. ≥pT3-stage

**Table:** 

A. pT3a-stage								
**LCC (mm)**	**no pT3a;** ***n***	**pT3a;** ***n***	**Total;** ***n***	**Cut-off**	**Sensitivity [95% CI]**	**Specificity** **[95% CI]**	**PPV** **[95% CI]**	**NPV** **[95% CI]**
**0–11**	33	17	50					
**12–17**	39	29	68	≥12	0.78 [0.68–0.86]	0.37 [0.27–0.47]	0.51 [0.42–0.60]	0.66 [0.52–0.78]
**18–23**	10	16	26	≥18	0.40 [0.30–0.51]	0.80 [0.71–0.87]	0.63 [0.49–0.76]	0.61 [0.52–0.70]
**≥24**	8	15	23	≥24	0.20 [0.12–0.29]	0.91 [0.84–0.96]	0.65 [0.45–0.82]	0.57 [0.49–0.65]
**Total**; ***n***	90	77	167		**AUC** 0.62 [95% CI 0.54–0.71]			
**B. ≥pT3b-stage**								
**Risk category**	**<pT3b;** ***n***	**≥pT3b;** ***n***	**Total;** ***n***	**Cut-off**	**Sensitivity** **[95% CI]**	**Specificity** **[95% CI]**	**PPV** **[95% CI]**	**NPV** **[95% CI]**
**LR**	67	5	72					
**L-IR**	66	12	78	≥L-IR	0.91 [0.82–0.97]	0.40 [0.33–0.48]	0.34 [0.27–0.42]	0.93 [0.86–0.98]
**H-IR**	33	30	63	≥H-IR	0.70 [0.73–0.85]	0.80 [0.73–0.85]	0.53 [0.42–0.65]	0.89 [0.83–0.93]
**HR**	1	9	10	≥HR	0.16 [0.08–0.27]	0.99 [0.97-1.00]	0.90 [0.63–0.99]	0.74 [0.67–0.80]
**Total;** ***n***	167	56	223		**AUC** 0.79 [95% CI 0.72–0.86]			
Risk categories for ≥pT3b. LR, L-IR-, H-IR or HR in case of 0, 1, 2, or 3 positive criteria, respectively: miT3b-stage (visual), LCC ≥18mm, PSMA-vol ≥7cm^3^								
**C. ≥pT3-stage**								
**Risk category**	**pT2;** ***n***	**≥pT3;** ***n***	**Total;** ***n***	**Cut-off**	**Sensitivity** **[95% CI]**	**Specificity** **[95% CI]**	**PPV** **[95% CI]**	**NPV** **[95% CI]**
**LR**	45	27	72					
**IR**	33	52	85	≥IR	0.80 [0.72–0.86]	0.50 [0.40–0.60]	0.70 [0.63–0.77]	0.63 [0.51–0.73]
**HR**	12	54	66	≥HR	0.41 [0.32–0.49]	0.87 [0.79–0.93]	0.82 [0.71–0.90]	0.50 [0.42–0.58]
**Total;** ***n***	90	133	223		**AUC** 0.70 [95% CI 0.63–0.77]			
Risk categories for ≥pT3. LR, IR-, or HR in case of 0, 1, or 2 positive criteria, respectively: LCC ≥18mm, PSMA-vol ≥3.3cm^3^								

LR = low risk; IR = intermediate risk; L-IR = low-intermediate risk; H-IR = high-intermediate risk; HR = high risk; Risk calculated as *n* of pathological T3a/≥T3b/≥T3 cases divided per total *n* of cases within the specified risk category.

The original article has been corrected.

The original article can be found at 10.1007/s00259-025-07757-3.

